# Clinical Characteristics of Hydrocephalus Following the Treatment of Pyogenic Ventriculitis Caused by Multi/Extensive Drug-Resistant Gram-Negative *Bacilli, Acinetobacter Baumannii*, and *Klebsiella Pneumoniae*

**DOI:** 10.3389/fsurg.2022.854627

**Published:** 2022-05-03

**Authors:** Sajan Pandey, Pei Wen Yao, Zhouqi Qian, Tao Ji, Ke Wang, Liang Gao

**Affiliations:** ^1^Department of Neurosurgery, Shanghai Tenth People's Hospital, Tongji University School of Medicine, Shanghai, China; ^2^Medical Department, Nanjing Medical University, Nanjing, China

**Keywords:** multiloculated hydrocephalus, uniloculated hydrocephalus, ventriculitis/meningitis, polymixin, intraventricular irrigation

## Abstract

**Objective:**

Hydrocephalus is common after ventriculitis. This study explores hydrocephalus's clinical characteristics following pyogenic ventriculitis due to multidrug-resistant and extensively drug-resistant *Acinetobacter baumannii* and *Klebsiella pneumoniae*.

**Patients and Methods:**

We retrospectively reviewed patients with post-neurosurgical pyogenic ventriculitis due to multidrug-resistant and extensively drug-resistant *A. baumannii* and *K. pneumoniae* in our department between January 2014 and June 2020. Once diagnosed, patients received intraventricular lavage followed by daily intraventricular administration of Colistin (polymyxin-E). The patient's clinical/radiographic findings were analyzed and evaluated 6 months after discharge.

**Results:**

In total, 48 cases were included in this study, and 25% were female. The median age was 45 (SD ± 15) years old. Median intraventricular Colistin administration to acquire sterile cerebrospinal fluid (CSF) was 20 days. Forty-one patients developed hydrocephalus; among them, 18 (43%) had multiloculated hydrocephalus (MLH), 23 (56%) had uni/non-loculated hydrocephalus (ULH/NLH), and 7 (17%) did not develop hydrocephalus. The patients with MLH had (15 days) delayed initiation of intraventricular irrigation (*p* < 0.022). They had (32 days) longer intraventricular Colistin (*p* < 0.003) and showed worse outcomes in terms of Glasgow outcome score (GOS) at 6 months follow-up than those without hydrocephalus. The mean score of the MLH group was 1.67 (SD1.23), and ULH/NLH was 2.61 (SD1.4) at *p* < 0.008.

**Conclusion:**

Multiloculated hydrocephalus is common in patients receiving delayed intraventricular administration of Colistin and required a longer duration on intraventricular Colistin to treat the pyogenic ventriculitis caused by multidrug/extensive drug-resistant *A. baumannii* and *K. pneumoniae*. It is associated with worse clinical outcomes.

## Introduction

Post-neurosurgical pyogenic ventriculitis is a severe complication that results in increased morbidity, mortality, and prolonged hospitalization (1). Its incidence after craniotomy is reported to be 1.5–5.5% (2, 3) and rises to 9.6–15% if external ventricular drainage is placed (4–6). The infection caused by multidrug-resistant or extensively drug-resistant (MDR/XDR) Gram-negative bacilli such as *Acinetobacter baumannii (AB) and Klebsiella pneumoniae (KP)* is challenging because of limited therapeutic measures (7). Intraventricular (IVT) administration of antibiotics is suggested to treat this condition (8). Our previous study on ventriculitis has shown an approximately 84% cure rate after initial IVT lavage followed by IVT administration of Colistin (9). However, despite the cure, these patients showed poor outcomes due to complicated hydrocephalus.

Patients can develop hydrocephalus following pyogenic ventriculitis. Uncontrolled hydrocephalus leads to increased intracranial pressure and even brain herniation. Usually, hydrocephalus demands a surgical intervention such as external ventricular drainage or lumbar drainage. Until now, there is little knowledge regarding the management of ventriculitis due to XDR/MDRAB/KP and its associated complication. This study investigated complex hydrocephalus (multiloculated/uniloculated) as a clinical complication of XDR/MDR ventriculitis following its successful treatment. The management strategy for uni/multiloculated hydrocephalus is beyond the scope of this study and would be better discussed separately.

### Case Illustration

“On 16^th^ Dec 2020”

**Figure 1:**
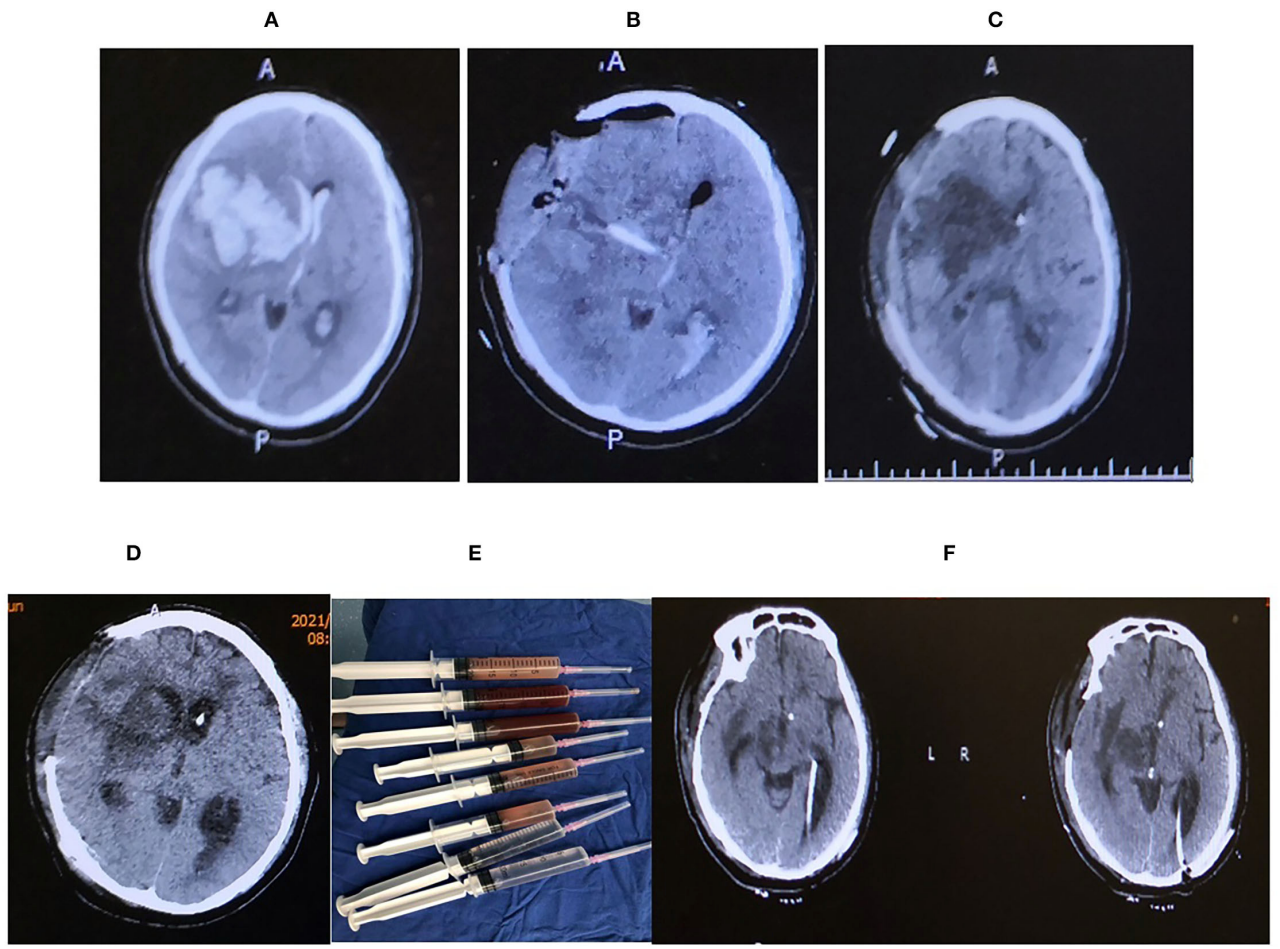
**(A)** Axial CT showing massive right hemisphere intracranial hemorrhage (ICH) extending to the ventricle with a mid-line shift toward left. **(B)** The hematoma was evacuated following decompressive craniotomy with intracranial pressure (ICP) probe and external ventricular drainage (EVD) *in situ*. **(C)** Ommaya pump was placed. **(D)** Axial CT head showing features suggestive of ventriculitis, **(E)** irrigation syringe following a thorough ventricular irrigation in the operating room, irrigation was done until the ventricular fluid becomes transparent, **(D)** Axial head CT after the ventricular irrigation and B/L EVD placement. **(F)** Axial CT head before discharge.

A 49-Years-old male presented with a chief complaint of sudden headache associated with a left-sided weakness for 3 h. He had a past medical history of hypertension and diabetes mellitus, ankylosing spondylitis, and lymph node tuberculosis. His Glasgow coma scale (GCS) gradually decreased to comatose, with dilated and fixed right pupil and pin-like left pupil. The initial head computer tomography (CT) scan showed right frontal intracranial hemorrhage (ICH) involving the basal ganglia, thalamus, to the ipsilateral ventricle (Figure 1A). He had emergent hematoma evacuation, decompressive craniotomy, and intracranial pressure (ICP) monitoring system implantation (Figure 1B). The postoperative period was uneventful until the second procedure of Ommaya pump implantation on the 13th day after the first operation due to obstructive hydrocephalus (Figure 1C). Despite antibiotic prophylaxis, he developed a high fever and was diagnosed with ventriculitis due to MDR*KP* on the 9th day after the second operation (Figure 1D).

**Figure 2:**
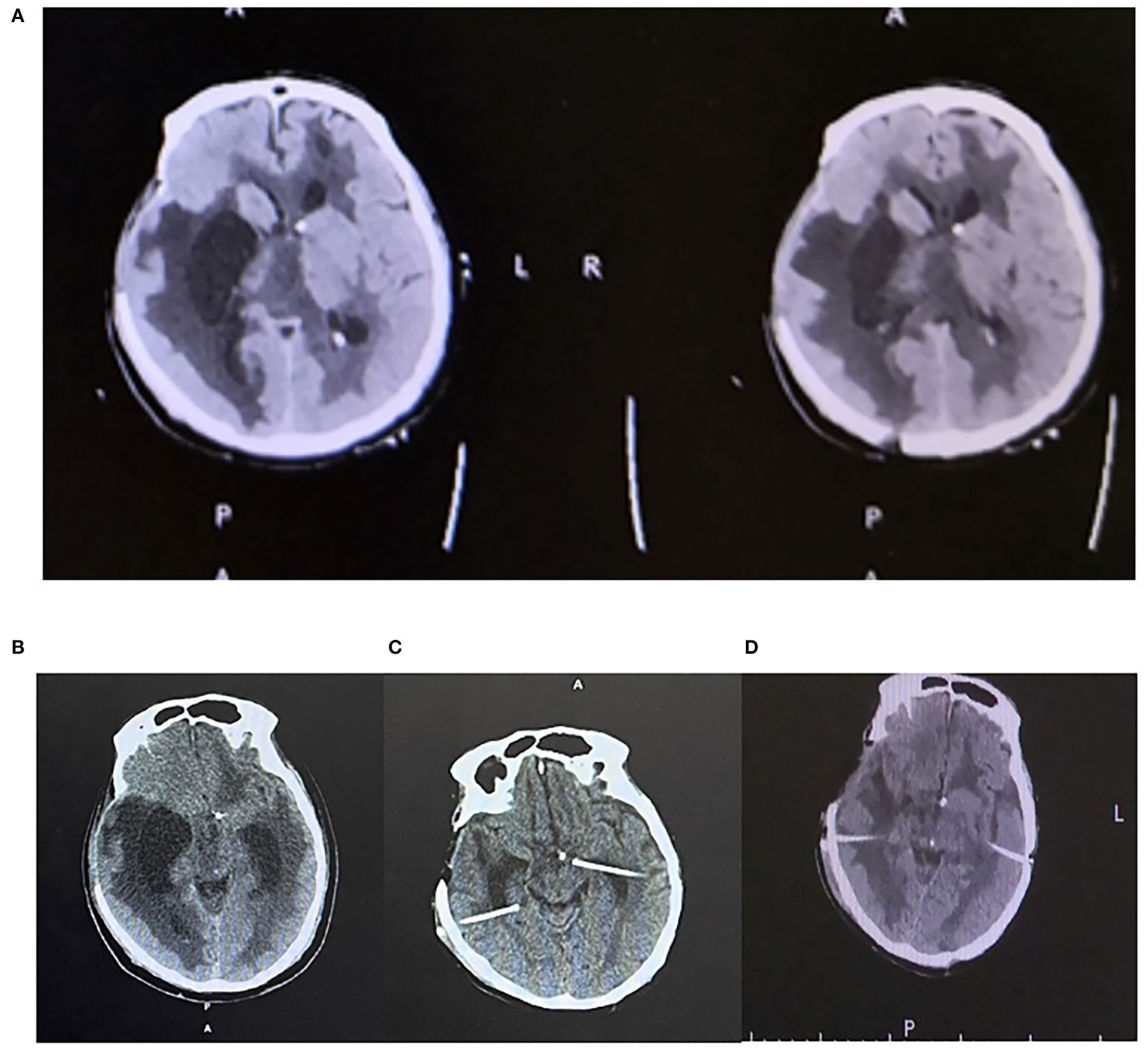
**(A)** Axial plane CT head showing multiple loculations in the ventricle. **(B)** Axial CT head showing enlarged ventricles prior to B/L shunt. **(C)** Axial head CT showing ommaya pump placement on B/L temporal horns. **(D)** Axial head CT after B/L VP shunt creation.

Ventricular irrigation with bilateral external ventricular drainage (EVD) was placed at the occipital horns followed by daily intraventricular administration of Colistin (Figures 1E,F), which lasted for 21 days until a sterile cerebrospinal fluid (CSF) sample was obtained. Although the ventriculitis was cured, the head CT showed multiple loculations with bilateral dilated temporal horns in the ventricle system. He received a ventriculoperitoneal shunt with two catheters placed in bilateral temporal horns and connected to an adjustable valve by a Y-shape connector. After the surgery, he survived and was discharged to a rehabilitation center (Figures 2A–D).

## Materials and Methods

### Study Design

This study was approved by the research and ethics committee of a hospital affiliated with a university. Written informed consent was obtained from the [individual(s) AND/OR minor(s)' legal guardian/next of kin] for the publication of any potentially identifiable images or data included in this article. A retrospective study was performed to explore the clinical characteristics of hydrocephalus following pyogenic ventriculitis due to MDR/XDRAB and KP.

### Selection

Data from patients with MDR/XDR ventriculitis admitted to our center between January 2014 to December 2020 were considered for the study. Inclusion criteria for the study were (i) every case with clinical and imaging evidence suggestive of ventriculitis, (ii) microbiological evidence of CNS infection, (iii) Isolation of MDR/XDR Gram-negative bacilli from the CSF, (iv) ventricular irrigation with Colistin mixed with normal saline in the operating room, and (v) IVT administration of Colistin.

Before admission, patients with severe systemic diseases, including chronic heart failure, respiratory insufficiency, liver and renal dysfunction, were excluded.

### Imaging

Uni/multiloculated hydrocephalus (ULH/MLH) is defined by the presence of isolated CSF compartments or compartments due to the presence of intra-ventricular septation seen in CT/MR imaging (10–12).

### Procedure

Lumbar puncture was performed immediately on patients with clinical signs and symptoms of bacterial meningitis. A CSF specimen was obtained for cellular/biochemical analysis, gram stain, culture, and sensitivity.

Patients with XDR/MDR ventriculitis were managed by inserting an external ventricular drainage system 5 cm from the skin to bone and 5.5 cm from the bone to the ventricle with Medtronic TM I. About 5 ml purulent specimen was aspirated before the irrigation for microbiological analysis. Irrigation was performed by adding 10 mg Colistin per 500 ml normal saline until the fluid became transparent.

### Antimicrobial Therapy

Empirical antibacterial therapy with vancomycin plus third-generation cephalosporin or vancomycin plus meropenem was initiated for suspected nosocomial bacterial meningitis (NBM). Following pathogen isolation, antibiotic treatment would be modified for optimal management according to susceptibility test (8). Ventriculitis cases due to *KP*/*AB* sensitive to Colistin was immediately switched to intravenous Colistin at 2,000,000 IU q 8 h and planned for immediate ventricular irrigation and daily IVT Colistin. If required, additional intravenous antibiotics were added for infections other than ventriculitis following the culture and sensitivity report.

### Minimal Inhibitory Concentration Evaluation

Minimal inhibitory concentration to polymyxin-E was measured with the broth microdilution method. The isolated pathogens and antimicrobial susceptibility testing showed susceptibility to Colistin. The MICs for KP and AB were <0.5 mg/L. The CSF concentrations of Colistin were continuously above 0.5 mg/L in all patients when they received Colistin at 100,000 units/day dose.

The following criteria were applied to ensure treatment until the patients were cured: (13) (a) white blood cell count in the CSF of < 20 cells/L for three consecutive tests and (b) two consecutive negative CSF cultures.

### Cerebrospinal Fluid Parameters for Ventriculoperitoneal Shunt Insertion

The following CSF parameters should meet the criteria before VPS: WBC < 20, RBC < 1,000, protein level < 1,200 mg/L, and sugar > 2/3rd of blood sugar level.

The intraventricular Colistin was administered at a dose of 100,000 IU (international unit) every 24 h. After IVT Colistin administration, CSF drainage was stopped for 2 h. Please refer to our previous study (Pharmacokinetics of Colistin in Cerebrospinal Fluid after Intraventricular Administration Alone in Intracranial Infections) for further details on administration (13).

### Patient Information

The following retrospective data were obtained from medical records: age, sex, underlying medical condition, isolated organism, delay in intrathecal therapy, type of antibiotics, antibiotic duration, presence of hydrocephalus and its subtypes according to morphology, and Glasgow outcome scale (GOS). Risk factors for nosocomial meningitis were categorized as follows: 1) history of neurosurgery, 2) CSF leakage or recent head trauma, and 3) distant focus of infection. The outcome was defined as a cure if a patient fulfilled the following criteria: (i) resolution of symptoms and signs of CNS infection, (ii) no focus of infection on enhanced MRI images, and (iii) eradication of MDR/XDR Gram-negative bacilli in subsequent CSF cultures. GOS was defined as follows: 1 = death, 2 = neurovegitative state, 3 = severe disability, 4 = moderate disability, and 5 = good recovery (14).

### Data Analysis

Continuous data were expressed as mean ± standard deviation (SD), and categorical data were expressed as median (interquartile range, IQR) or percentage. Statistical analyses were performed with SPSS 26.0 for Windows. The *p*-values were derived from two-tailed unpaired Student's *t*-test, Mann–Whitney test, Pearson's chi-square test, or Fisher's exact test. Logistical regression analysis was performed to adjust the effects of confounders. Differences were considered significant if the *p*-value was below 0.05.

## Results

We enrolled 48 patients meeting the required criteria. The mean age was 44 SD ± 15 years old and 25% were female. Among the 48 patients, 41 (85.4%) had hydrocephalus, 18 (37.5%) had MLH, and 23 (47.9%) had uni/non-loculated hydrocephalus (ULH/NLH). Patient demographics are summarized in Table 1.

**Table 1:** Shows the gross demographic distribution of patients with and without hydrocephalus; age, gender, previous surgery, isolated organism, and source of infection.

	**Hydrocephalus (n 41) MLH 18 (43.9%) ULH 23 (56.1%)**	**No hydrocephalus (n 7)**	***P*-value**
Age in years	44.05 (SD 16.15)	45.29 (SD 13.18)	0.2
Gender (Male)	32 (78%)	4 (57.1%)	0.34 Fisher
Previous surgery			0.88
TBI	25 (61%)	4 (57.1%)	
ICH	15 (36.6%)	3 (42.9%)	
Tumor	1 (2.4%)	0	
Organism			0.32 Fisher
Accinobacter baumannii	33 (80.5%)	4 (57.1%)	
Klebsiella pneumonia	8 (19.5%)	3 (42.9%)	
Infection source			0.81
Surgical wound infection	13 (31.7%)	3 (42.9%)	
Lumbar Drain	9 (22%)	1 (14.3%)	
External ventricular drain	19 (46.3%)	3 (42.9%)	

*MLH, Multilobulated hydrocephalus; ULH, Unilobulated hydrocephalus*.

Delayed intraventricular irrigation and treatment were observed in 38 out of the 48 patients. Intrathecal treatment delay with sensitive antimicrobial after isolation of MDR/XDR organism was 8 (SD ± 6) days for the 7 patients without hydrocephalus, 11 (SD ± 6) days for the 23 patients with ULH/NLH, and 15 (SD ± 5) days for the 18 patients with MLH.

The patients with MLH had significantly longer delayed days than those with ULH/NLH (15 ± 6 vs. 11 ± 6, *p* = 0.022). The patients with ULH/NLH had longer delayed days than those without hydrocephalus, but the differences were not significant enough (11 ± 6 vs. 8 ± 6, *p* = 0.127) (summarized in Tables 2, 3).

**Table 2:** Comparison between Multiloculated vs. uni/non-loculated hydrocephalus (MLH/ULH/NLH) in terms of delay in intraventricular treatment in days, Glasgow outcome score (GOS).

	**MLH n18 (37.5%)**	**ULH/NLH n23(47.9%)**	***p***
**IVT delay in days**	15 (SD ± 5)	11 (SD ± 6)	**0.022**
**IVT duration in days**	32 (SD ± 15)	26 (SD ± 14)	**0.003**
**GOS**	1.67 (SD ± 1.23)	2.61 (SD ± 1.4)	**0.008**
Dead	13 (72.2%)	8 (34.8%)	
Vegetative	1 (5.6%)	3 (13%)	
Lower Sev disability	2 (11.1%)	5 (21.7%)	
Upper Sev disabilty	1 (5.6%)	4 (17.4%)	
Lower mod disability	1 (5.6%)	3 (13%)	

*There was a significant difference among two groups at p < 0.05*.

*P value < 0.05 was considered significant and were shown in bold*.

**Table 3:** Comparison between Uni/non-loculated hydrocephalus (ULH/NLH) vs. with out hydrocephalus in terms of Delay in intraventricular treatment, intraventricular treatment in days, Glasgow outcome score (GOS).

	**ULH/NLH n23**	**No hydrocephalus n7**	***p***
**IVT delay in days**	11 (SD ± 6)	8 (SD ± 6)	0.127
**IVT duration in days**	26 (SD ± 14)	13 (SD ± 10)	**0.041**
**GOS**	2.61 (SD ± 1.4)	3.29 (SD ± 1.6)	0.08
Dead	8 (34.8%)	1 (14.3%)	
Vegetative	3 (13%)	2 (28.6%)	
Lower Sev disability	5 (21%)	0	
Upper Sev disability	4 (17.4%)	2 (28.6%)	
Lower mod disability	3 (13%)	2 (28.6%)	

*Rank test shows there was a significant difference among two groups in terms of intraventricular treatment duration and GOS at p < 0.05*.

*P value < 0.05 was considered significant and were shown in bold*.

The average intraventricular treatment day for MLH was 32 (SD ± 15), 26 (SD ± 14) for ULH/NLH, and 13 (SD ± 10) for the patients without hydrocephalus. The patients with MLH required more days of intrathecal treatment than the other groups. Their mean difference was significant at *p* = 0.003. The data show that the ULH/NLH group required more days of intrathecal therapy than the patients without hydrocephalus. The mean difference was significant at *p* = 0.041.

The mean GOS score of the MLH group was 1.67 (SD 1.23), ULH/NLH was 2.61 (SD1.4), and non-hydrocephalus was 3.29 (SD1.6). Thirteen (72.2%) patients from the MLH group died, 1 (5.6%) remained vegetative, 2 (11.1%) had a lower severe disability, 1 (5.6%) had an upper severe disability, and 1 (5.6%) had a lower moderate disability. Eight (34.8%) patients in the ULH/NLH group died, 3 (13%) remained vegetative, 5 (21.7%) had a lower severe disability, 4 (17.4%) had an upper severe disability, and 3 (13%) had a lower moderate disability. The Rank test showed a significant difference between MLH and without, which means the patients with MLH had a significantly lower GOS score than those without at *p* = 0.008. However, the difference between the ULH/NLH and without hydrocephalus was insignificant at *p* = 0.08 (Tables 2, 3).

A total of 10 patients from the MLH group had permanent shunt surgery for hydrocephalus, 9 (90%) required two intraventricular catheters, whereas 1 case required a single catheter, while in the ULH/NLH group, 17 (94%) required a single intraventricular catheter, and one required double catheters.

## Discussion

Ventriculitis due to XDR/MDR Gram-negative bacilli is rare. Despite several standard precautions (i.e., maintaining hand hygiene, strict monitoring of compliance, contact barrier precautions, environmental and tool disinfection protocols, use of prophylactic antibiotics, invasive procedures under aseptic conditions, routine wound care, avoiding prolonged EVD, use of antibiotic-coated EVD, measures to reduce catheter colonization, and adequate nutrition), we still come across these organisms. These are associated with significant morbidity and mortality due to drug resistance or inadequate antibiotic penetration through the blood-brain barrier (BBB) (15, 16). IVT Colistin administration through an external ventricular drainage system following irrigation with 10 mg Colistin per 500 ml normal saline until the irrigation fluid became transparent would bypass the penetration effect due to BBB. It may also reduce the inflammatory process by washing away inflammatory materials and improve CSF flow by diluting exudates (17). This therapeutic approach has an 84% cure rate (17), but 16% of patients did not respond to the treatment. Could it be due to complications following ventriculitis?

We observed that cases of hydrocephalus following XDR/MDR were extremely high. A total of 41 (85.4%) out of the 48 patients developed hydrocephalus. Among them, 18 (37.5%) had MLH, and 23 (47.9%) had ULH/NLH. Since our sample size predominates adults, the primary factor requiring surgery in our series was TBI 62.5% and ICH 35.4%. A total of 62% of the patients developed an infection from the EVD and lumbar drain, 33.3% from the surgical wound site, and 4% from CSF leakage complications. The common risk factors for loculations are intracerebral hemorrhage (ICH), bacterial meningitis, over-drainage of CSF, and foreign bodies such as shunts that were placed (18–21). These loculations are very difficult to treat (18). Because of loculations, the fluid cavity keeps enlarging as it cannot drain. Enlarging cysts themselves or enlarging cysts obstructing the foramen of Monro could lead to hydrocephalus (11, 22, 23).

### Delayed Treatment and MLH

Our study suggests that delayed initiation of IVT irrigation and administration of antibiotics led to a higher incidence of MLH. The independent sample *t*-test performed on patients with and without MLH showed a significant difference at *p* < 0.02. At the same time, the test between ULH/NLH and without hydrocephalus was insignificant (*p* = 0.127), which could be due to the smaller sample size of the non-hydrocephalus group.

### Intraventricular Septation/Loculation

The possible rationale behind the loculation in these patients can be explained based on the poor penetration of intravenous antibiotics through the BBB (24). The minimal effect of the antibiotics promotes a suitable environment for further bacterial proliferation. Increased proliferation and inflammation further raise viscosity because of exudates and debris (25). These accumulated exudates and inflammatory debris promote IVT septation from the microglial membrane (25).

According to a previous literature review (26), polymyxins themselves can cause chemical ventriculitis, which we did not observe in our study. A case report by Guo et el. (27) reported transient ventricular adhesion following IVT administration of polymyxin-B. Could this phenomenon further promote loculation? If other antibiotics (sensitive) were used, could the incidence of MLH not be so high? We do not know yet, which certainly demands further study.

The delayed initiation of IVT lavage and administration of Colistin in our study was mainly due to a delay in referral to our center. Even after the isolation of XDR/MDR bacteria, referring centers kept these patients on intravenous polymyxin and other antibiotics. All incoming positive-culture XDR/MDR cases were immediately taken to OR for IVT irrigation and treatment.

### Treatment Duration and Outcome

Our study showed that the MLH group required six more days of IVT Colistin than the ULH/NLH group at *p* < 0.003. Similarly, ULH/NLH required 13 more days than NLH at *p* < 0.041. To explain this, we should understand the anatomy inside the ventricle after loculation. We hypothesize that the loculation process itself hampers the distribution of antibiotics. Once loculation is thoroughly established, it acts as an isolated compartment where antibiotics cannot diffuse, unlike in patients without loculation. The slower antibiotic response across the loculi could be the possible reason for the prolonged sterilization period. The extent of penetration could differ across each loculus, resulting in variance in sterilization duration in patients with single or multiple loculations. Since IVT administration is associated with chemical ventriculitis and transient ventricular adhesion, prolonged exposure might complicate loculation.

We also found that the GOS score was significantly low among the MLH group, as in previous reports (10). Since our population was adults except for prematurity, the common cause of death was bacterial infection (28). Our MLH cases also required more shunt surgeries than the ULH/NLH cases because of shunt failure (22, 23). Furthermore, among the patients with MLH who survived the infection, 90% required two ventricular catheters (connected to a single valve by Y connector) and a permanent shunt to control the hydrocephalus, whereas 94% of the patients with ULH/NLH were managed with a single catheter. Repeated shunting explains how each cavity acts as an isolated compartment that mandates restoring communication. Persistent infectious state and increased intracranial pressure may precipitate irreversible brain injury (28), resulting in a low GOS score among these groups.

There are several limitations to our study. First, it is a retrospective analysis with the possibility of unmeasured bias. Second, due to the rarity of this condition, our sample size is small. We need a larger sample size for accurate prediction. Despite these limitations, this study suggests that patients with MDR/XDR ventriculitis sensitive to Colistin should be treated early by IVT irrigation and Colistin administration. Delayed treatment was significantly associated with a high risk of development of MLH and ULH, requiring a longer duration of IVT administration to sterilize CSF, and associated with a low GOS score.

## Conclusion

Ventriculitis due to XDR/MDR is rare and difficult to treat. It is associated with a high risk of hydrocephalus. Delay in appropriate therapeutic approaches for these patients increases the incidence of complicated hydrocephalus (MLH and ULH). MLH and ULH are associated with delayed and longer duration of IVT antibiotic administration, multiple shunt failures, and low GOS score.

## Data Availability Statement

The raw data supporting the conclusions of this article will be made available by the authors, without undue reservation.

## Ethics Statement

The studies involving human participants were reviewed and approved by Ethics and Review Committee of Shanghai Tenth People's Hospital, Tongji University School of Medicine, Shanghai, China. The patients/participants provided their written informed consent to participate in this study.

## Author Contributions

SP: manuscript writing, data analysis, and reviewing manuscript. SP and PY: data collection and writing manuscript. ZQ: data collection. TJ: study design, data, and manuscript review. KW: data analysis, manuscript writing and reviewing, and proof reading. LG: study design and proof reading. All authors contributed to the article and approved the submitted version.

## Conflict of Interest

The authors declare that the research was conducted in the absence of any commercial or financial relationships that could be construed as a potential conflict of interest.

## Publisher's Note

All claims expressed in this article are solely those of the authors and do not necessarily represent those of their affiliated organizations, or those of the publisher, the editors and the reviewers. Any product that may be evaluated in this article, or claim that may be made by its manufacturer, is not guaranteed or endorsed by the publisher.
