# Erratum on: Clinical Characteristics of Hydrocephalus Following the Treatment of Pyogenic Ventriculitis Caused by Multi/Extensive Drug-Resistant Gram-Negative *Bacilli, Acinetobacter Baumannii*, and *Klebsiella Pneumoniae*

**DOI:** 10.3389/fsurg.2022.971727

**Published:** 2022-07-26

**Authors:** Frontiers Production Office

**Affiliations:** Frontiers Media SA, Lausanne, Switzerland

**Keywords:** multiloculated hydrocephalus, uniloculated hydrocephalus, ventriculitis/meningitis, polymixin, intraventricular irrigation

An Erratum on Clinical Characteristics of Hydrocephalus Following the Treatment of Pyogenic Ventriculitis Caused by Multi/Extensive Drug-Resistant Gram-Negative Bacilli, Acinetobacter Baumannii, and Klebsiella Pneumoniae by Pandey S, Yao PW, Qian Z, Ji T, Wang K, and Gao L. (2022). Front. Surg. 9:854627. doi: 10.3389/fsurg.2022.854627

Due to a production error in the published article, a sentence in the Introduction section has been edited incorrectly. The sentence reading “These patients refer (patients from our previous study whose infection was cured by our treatment protocol but the functional status remained poor)” has been corrected to: “However, despite the cure, these patients showed poor outcomes due to complicated hydrocephalus.”

This error does not change the scientific conclusions of the article in any way. The publisher apologizes for this mistake.

The original article has been updated.

## Publisher's Note

All claims expressed in this article are solely those of the authors and do not necessarily represent those of their affiliated organizations, or those of the publisher, the editors and the reviewers. Any product that may be evaluated in this article, or claim that may be made by its manufacturer, is not guaranteed or endorsed by the publisher.

